# Clinical Efficacy and Safety of Benjakul Remedy Extract for Treating Primary Osteoarthritis of Knee Compared with Diclofenac: Double Blind, Randomized Controlled Trial

**DOI:** 10.1155/2017/9593580

**Published:** 2017-10-12

**Authors:** Patamaporn Rachawat, Piya Pinsornsak, Puritat Kanokkangsadal, Arunporn Itharat

**Affiliations:** ^1^Department of Applied Thai Traditional Medicine, Faculty of Medicine, Thammasat University, Pathum Thani 12120, Thailand; ^2^Department of Orthopedics, Faculty of Medicine, Thammasat University, Pathum Thani 12120, Thailand; ^3^Center of Excellence in Applied Thai Traditional Medicine Research (CEATMR), Thammasat University, Pathum Thani 12120, Thailand

## Abstract

**Background:**

The purpose of this study was to investigate the clinical efficacy and safety of Benjakul (BJK) extract for treating primary osteoarthritis (OA) of the knee compared with diclofenac.

**Methods:**

A phase 2, double blind, randomized, and controlled study was conducted. The BJK group received 300 mg of BJK extract per day, while another group received 75 mg of diclofenac per day. All patients were followed up at 14 and 28 days. The changing of visual analogue scale (VAS) for pain, 100-meter walking times, the modified Thai WOMAC index scores, and the global assessment were evaluated for efficacy. For safety issue, clinical signs and symptoms, complete physical examination, and renal and liver function were evaluated.

**Results:**

39 and 38 patients for BJK extract group and diclofenac group were evaluated. For efficacy, all patients from both groups reported a decrease in the VAS pain score and 100-meter walking times but only the diclofenac group showed significant reduction of both measurements when compared with day 0. The modified Thai WOMAC scores of both groups were significantly reduced from baseline. However, all efficacy outcomes were not significantly different for both groups. For safety outcomes, the patients from both groups had no severe adverse events reported and only BJK had no toxicity in renal and liver functions.

**Conclusions:**

The BJK remedy extract showed equal clinical efficacy in relieving symptoms of OA knee when compared with diclofenac.

## 1. Background

Nowadays, advancement of medical science gives people a longer live span. From 2015 to 2050, the proportion of the world's population over 60 years will nearly double from 12% to 22% [1]. The world population is becoming an aging society and its health problems are under surveillance. Osteoarthritis (OA) of the knee is one of the most common diseases in elderly people. OA is a degenerative disease of the joint surface resulting from inflammatory process. This process leads to damage of cartilage, entire joint, and surrounding soft tissue. The main symptoms of OA are knee pain on movement, stiffness, and functional limitation. Current OA treatment aims to relieve pain, limit progression of joint damage, and improve functional status [2]. Oral medication especially, nonsteroidal anti-inflammatory drugs (NSAIDs), is commonly used for treatment of OA. NSAIDs inhibit cyclooxygenase enzyme (COX) which synthesizes prostaglandins as a cause of pain. However, they have some side effects such as peptic ulcer, liver, and renal dysfunction. Thus, there is keen interest in alternative medicines which seem to have less side effects.

Benjakul (BJK) is a Thai traditional medicine remedy that is composed with 5 plants: fruits of* Piper retrofractum* Vahl. (Piperaceae), roots of* Piper sarmentosum *Roxb. (Piperaceae), stem of* Piper interruptum Opiz. *(Piperaceae), roots of* Plumbago indica *L. (Plumbaginaceae), and rhizome of* Zingiber officinale *Roscoe. (Zingiberaceae). In southern Thai folk medicine, BJK is used as an adaptogenic drug [3]. There is evidence that the ethanolic extract of BJK showed anti-inflammatory activity by inhibitory effect on nitric oxide (NO) release (IC_50_ = 18.23 *μ*g/ml) and anti-free radical activity (EC_50_ = 14.9 *μ*g/ml) [4]. In an* in vivo* study, it also reduced inflammatory signs such as pain, edema, and fever in inflammatory animal models [5]. BJK extract seems to have efficacy similar to NSAIDs. Moreover, it has also shown no renal and liver toxicity in animal models [4] and has also shown the same safety results in chronic toxicity in animal model [4] and clinical trial phase 1, in healthy volunteers [6]. This data should be explored using BJK extract in treating OA of knee which has not been scientifically researched before. Thus, the purpose of this study was to investigate the clinical efficacy and safety of BJK extract for treating OA knee, compared with the standard NSAID, diclofenac.

## 2. Methods

### 2.1. Research Design

This study was a randomized, double blind, and controlled trial (phase 2), designed to study the clinical efficacy and safety of Benjakul remedy by comparison with diclofenac for the treatment of osteoarthritis of knee at Thammasat University Hospital, Pathum Thani, Thailand. This trial was approved by the Medical Ethics Committee of the Faculty of Medicine, Thammasat University. This committee was accepted by FDA Thai Government (registry number MTU-EC-ES-6-152/56) and also was registered at ClinicalTrials.gov (NCT02286453).

The sample size determination was calculated from this formula, *N* (each group) = (*Zα*/2 + *Zβ*)2*∗*2*∗σ*2/*d*2, where *Zα*/2 is the critical value of the Normal distribution at *α*/2 (e.g., for a confidence level of 95%, *α* is 0.05 and the critical value is 1.96), *Zβ* is the critical value of the normal distribution at *β* (e.g., for a power of 90%, *β* is 0.1 and the critical value is 1.28), *σ*2 is the population variance, and *d* is the difference you would like to detect. But this trial did not have any preliminary study data to calculate “*d*” value. Thus, my statistical consultant advised me to use 0.75 times standard deviation (*σ*) as minimum detectable difference in means based on “effect size” theory [7].

Thus, the minimum sample size for each group to detect the mean difference between the two means is 38 persons/group. Lastly, considering 10 percent of drop-out was count out, so forty-two patients per each treatment group were required for the study.

### 2.2. Subjects

Eighty-four outpatients, from the Department of Orthopedics, Thammasat University Hospital, aged between 45 and 80 years old, who were diagnosed with primary osteoarthritis of knee, based on American College of Rheumatology's clinical and radiological criteria with at least 20 mm arthritis pain rating of target knee [100 mm. visual analogue scales (VAS)] were recruited [8]. The patients rated with severe osteoarthritis of knee (Kellgren–Lawrence radiographic system grade 4 at the time of screening), patients with serious medical conditions such as uncontrolled hypertension (BP > 140/90 mm·Hg), severe gastrointestinal (GI) disease, congestive heart disease, and renal or liver dysfunction, and obese patients assessed by body mass index (BMI) more than 32 kg/m^2^ were excluded from this study [9].

### 2.3. Drug Preparation

The parts of plants in Benjakul (BJK) remedy were collected from Kanchanaburi province (Table 1), Thailand. All plants were cleaned immediately of extraneous material, dried at 50°C., weighted into equal proportions, mixed together and ground to be coarsely powdered, then macerated at room temperature with 95% ethanol for 3 days, and filtered through a Whatman number 1 filter paper. The residue was further macerated with the same solvents two more times. The extracts were concentrated by using a rotary evaporator (Rotavapor R-205, Buchi, Switzerland) and dried by lyophilizer.

Finally, the extract was made to be powder and contained in 500 mg capsule (the concentration of BJK extract is 100 mg per capsule). All plant ingredients and BJK preparation were quality controlled based on Thai herbal pharmacopeia (appearance, chemical fingerprints, disintegration, microbial contamination, heavy metal contamination, and loss on drying).

The BJK extract capsule was also tested for stability by accelerated shelf life testing (ASLT) method. Chemical stability was tested by high-performance liquid chromatography (HPLC), using piperine as biomarker. Piperine content in BJK extract was determined as 80.76 mg/g. Meanwhile, biological stability was tested by inhibitory effect on nitric oxide release activity on RAW 264.7 murine macrophage cell lines [4]. Diclofenac sodium, 25 mg enteric-coated tablets (Voltaren®), is composed of benzene-acetic acid derivative for oral administration. Voltaren was manufactured and distributed by Novartis pharmaceutical Corp. (Thailand) and encapsulated in the same size and colour as BJK extract capsule. Omeprazole (20 mg) (Miracid, Berlin) was used as an open labeled medication.

### 2.4. Procedure

The patients who met the inclusion criteria were informed, signed in consent form, and divided randomly to either the BJK extract treatment group or diclofenac treatment group using computer generated program by individual who did not contact all investigators involved in trial. Each of the patients received the same appearance treatment with a randomized code number sequentially from a secret random list which was not revealed until data collecting was completed or medical emergency developed. All treatment assignment was also concealed to all investigator involved in trial too.

In the clinical trial, demographic data, clinical signs and symptoms, complete physical examination, laboratory test (complete blood count (CBC), lipid profile, liver function test, renal function test, and urine analysis), visual analogue scale (VAS) for pain, 100-meter walk times, and the modified Thai WOMAC index scales were collected on the first visit for baseline data and again after receiving treatment on days 14 and 28.

### 2.5. Drug Administration

The patients were divided randomly into 2 treatment groups. The patients in group 1 received the BJK extract 300 mg/day (1 capsule of 100 mg BJK extract three times daily after meals). The patients in group 2 received diclofenac sodium 75 mg/day (1 capsule of 25 mg diclofenac three times daily after meals). In addition, the patients in both groups received 20 mg of omeprazole, 1 capsule before breakfast for prophylaxis of GI adverse effects.

The human dosage in this study was taken from that used in southern Thai folk medicine at 1000 mg/meals of BJK remedy in crude drugs powder form [3]. Yield of crude extract in this study was 11.16%, so when back-calculated from 100 mg of BJK extract to dose in crude drugs powder form, it was close to the traditional used dose. Moreover, it is the minimum dose of the BJK extract that was already evaluated for safety from clinical trial phase 1 [6].

*Assessment*. The treatment period was completed in 28 days with the clinical and laboratory investigation follow-up assessments at the 14th and 28th days. After treatment, the global assessment was performed by the patients at the last visit.

The clinical efficacy was evaluated from the VAS pain score, the 100-meter walking times, and the modified Thai WOMAC index score at day 0, day 14, and day 28. The modified Thai WOMAC index is a questionnaire that was modified from the latest version of WOMAC 3.1 and translated to Thai language for suitability for Thai culture. Index of content validity (ICV) ranged from 0.25 to 1.00 [7]. The global assessment on a 0–4 likert scale (0: none, 4: excellent) was evaluated on day 28. The safety outcomes were evaluated by clinical examination and laboratory investigation.

The toxicity of drug was considered for excluding patients followed by guidance for industry in toxicity grading scale of USFDA such as creatinine more than 1.7 mg/dL, BUN more than 26 mg/dL, AST and ALT more than 2.5 times upper limit of normal (ULN), or ALP more than 2.0 times ULN.

### 2.6. Statistical Analysis

The changes in the mean values from baseline to day 14 and day 28 for each group were analyzed by the repeated measured analysis of variance (ANOVA) or Friedman's test. The comparison of the mean values between the two groups was analyzed by Student's *t*-test or Mann–Whitney *U* test. The comparison of the global assessment report was analyzed by Chi-square test.

## 3. Results

**Figure 1:**
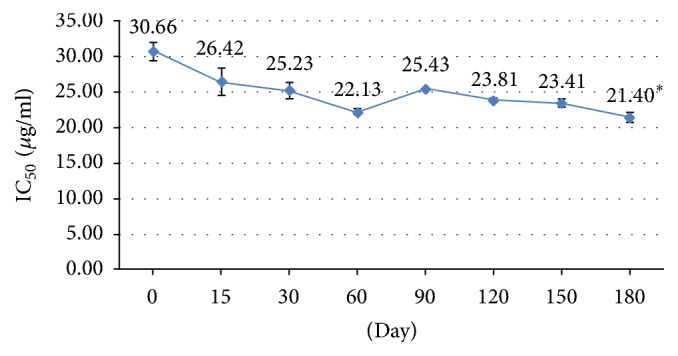
Biological stability test of BJK extract capsule: anti-nitric oxide (NO) release study. All data are mean ± SEM as obtained by triplicate analysis. ^*∗*^Significant difference from day 0 (*P* value < 0.05).

**Figure 2:**
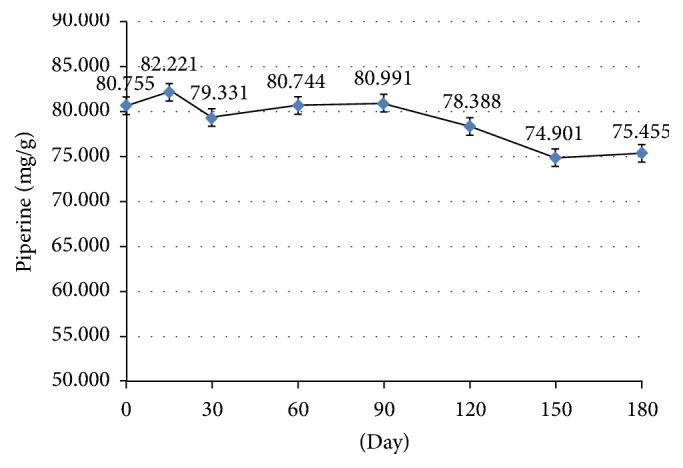
Chemical Stability test of BJK extract capsule: Piperine content of Benjakul extract. All data are calculated as standard linear equation: *Y* = 41.58*x* + 484.4, *R*^2^ = 0.999, where *Y* is peak area and *X* is the concentration of the analyzed sample.

The test for stability of biological activity of BJK extract capsules by inhibition of NO production found that IC_50_ at day 180 was significantly reduced from baseline, at 21.4 *μ*g/ml and 30.66 *μ*g/ml, respectively (Figure 1). It means that BJK extract at day 180 showed better anti-inflammatory effect than day 0. It is a good point for this effect. The chemical stability test found that piperine content of BJK extract capsules at day 180 was slightly reduced however it is not significantly different from baseline (day 0), at 75.46 mg/g and 80.76 mg/g, respectively (Figure 2). These results concluded that the BJK capsules had a shelf life of at least two years from the date of processing [10] which cover the trial period.

**Figure 3:**
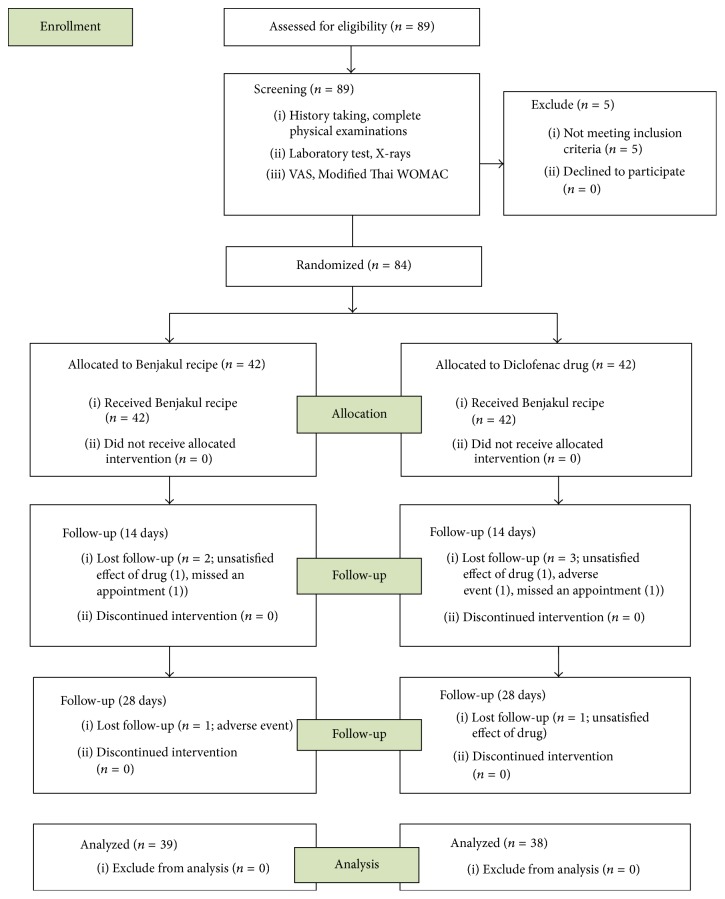
Flow chart of patients.

From the screening total of 89 patients, 5 patients were excluded from the study due to abnormal liver function tests. The eligible 84 patients were randomized into 2 groups (42 patients each group). Their baseline characteristics data (Table 2) and the radiographic disease grading (Table 3) between the two groups were not significantly different. Of the 84 patients, 77 of them completed the study (39 in BJK extract group and 38 in diclofenac group). Five patients dropped out at the first follow-up (2 patients were unsatisfied with efficacy of diclofenac and BJK, a patient suffered an adverse event of diclofenac, a patient was lost to follow-up in diclofenac group, and a patient was lost to follow-up in BJK group). Two patients dropped out at the last follow-up (1 patient unsatisfied with efficacy of diclofenac and 1 patient suffered an adverse event of BJK). The results are shown in Figure 3.

*(1) Efficacy*. Results from the study showed that both the BJK extract remedy and diclofenac reduced the VAS pain score but only diclofenac significantly reduced the mean of VAS pain scores on day 14 and day 28. However, there was no significant difference between the 2 groups. Both groups reduced the 100-meter walking times but BJK extract group was not significantly reduced from the baseline, while the diclofenac group was significantly reduced only on day 14. However, there was no significant difference between the 2 groups. For the modified Thai WOMAC index scores, all the 3 subscales (pain index, stiffness index, and physical function index) and total scores, the reduction in mean score occurred in a similar manner in both groups. The BJK extract group was significantly reduced on day 28 while that of the diclofenac group was significantly reduced on both day 14 and day 28. However, there were no significant difference between the BJK extract group and the diclofenac group (Table 4). At the end of study, the global assessment showed improvement of symptoms in both groups but had shown no significant difference between the 2 groups. The majority of the patients in both groups indicated scores of “moderately better” and “much better” (Table 5).

*(2) Safety*. The most adverse event found in BJK extract group was stomach pain (equally 7.14%). In diclofenac group, heartburn was the most common event at 9.52% (Table 6). However, there was no significant difference between the 2 groups. In both groups, the blood pressure measurement, both systolic and diastolic, was not significantly different from baseline and also not significantly different between the 2 groups (Table 7). For patient's safety, liver and renal functions were tested, blood urine nitrogen (BUN), creatinine for renal function tests, and AST, ALT, and ALP for liver function tests. In BJK extract group, all renal function tests showed no significant reduction while, in the diclofenac group, both BUN and creatinine levels were significantly increased from baseline. The renal function comparison between the 2 groups showed significant difference on day 14 and day 28 for BUN levels, while creatinine level was not significantly different between the 2 groups. For liver function tests, the BJK extract group showed slight increase but not significant increase in all AST, ALT, and ALP levels on day 14 and day 28, while the diclofenac group showed significant increase in all AST, ALT, and ALP levels on day 14 and day 28. The chemical liver function comparison between the 2 groups found that AST and ALP levels were not significantly different while AST level was significantly different on day 14. However, there was no significant difference of AST level on day 28. The changing in blood pressure, renal function, and liver function in both groups was in normal range. However, ALT on day 14 were significantly different between the 2 groups (*P* = 0.016). Diclofenac showed higher ALT value than BJK on day 14 (Table 7).

## 4. Discussion

Although the BJK remedy is the Thai traditional medicine which has been widely used over a long period to balance health, there is little documented research. In a previous report, BJK ethanolic extract showed good inhibitory effect on nitric oxide (NO) release as proinflammatory mediator in activated murine macrophages cell line (RAW 264.7) (IC_50_ = 18.23 *µ*g/ml) [4]. Piperine, which is a major compound in the ethanolic extract of BJK and is also a major compound of* Piper retrofractum *Vahl.,* Piper sarmentosum* Roxb., and* Piper interruptum Opiz.,* has shown anti-inflammatory activity on human OA chondrocytes [11] by inhibiting the IL-1*β* which induces the production of PGE2 and NO. In addition, the BJK ethanolic extract also showed anti-inflammatory activity in animal model. For example, the BJK extract reduced the development of rat ear edema induced by ethyl phenylpropiolate (EPP), reduced the development of rat paw edema induced by carrageenan, and also significantly reduced the amount of time spent licking in rat induced by formalin injection (formalin test) [5]. There is another research report of the plant ingredients of BJK which studied inflammatory activity. The ethanolic extracts of* Piper interruptum* Opiz. and* Piper retrofractum *Vahl. also showed anti-inflammatory, analgesic, and antipyretic activities in animal model. Both extracts inhibited ethyl phenylpropiolate-induced ear edema and carrageenan-induced hind paw edema in rats and reduced transudative and granuloma weights as well as body weight gain and thymus weight of the chronic inflammatory model using cotton pellet-induced granuloma formation in rats; they also exhibited analgesic activity on both early phase and late phase of formalin test in mice and also showed antipyretic activity on yeast-induced hyperthermia in rats [12]. Moreover, there are many studies of ginger (*Zingiber officinale *Roscoe) which also showed anti-inflammatory activity. The ethanolic extract of ginger showed good inhibitory effect on NO release in activated murine macrophages cell line (RAW 264.7) (IC_50_ = 11.93 *µ*g/ml) [13]. Its isolated compound from methanol extract, 8-shogaol and 10-shogaol, can inhibit COX-2 but not COX-1 with IC_50_ values of 17.5 *μ*M and 7.5 *μ*M, respectively [14]. In human study of ginger extract (EV ext-33; Eurovita Extract 33) 170 mg dose for three weeks showed relieve of pain in OA patients better than placebo but lower than ibuprofen (400 mg) [15]. And ginger extract (EV ext-77; Eurovita Extract 77) at 255 mg dose for three weeks showed relieve of pain on standing and after walking 50 feet in OA patients better than placebo [16].

Piperine content and IC_50_ value of BJK extract capsule from stability test indicate that BJK extract capsule has an acceptable 2-year shelf life for this study. In previous study, piperine from the ethanolic extract of BJK has been shown to be a stable compound in accelerated conditions (45 ± 2°C with 75 ± 5% RH for 4 months) [17]. This study confirms that piperine content from BJK extract capsule is also stable in accelerated conditions (40 ± 2°C with 75 ± 5% RH for 6 months). However, IC_50_ of anti-inflammatory effect of BJK extract capsule in accelerated conditions shows improved anti-inflammatory efficacy in contrast to the reduction of piperine in our study. This contrast of both IC_50_ and piperine data possibly arises from synergistic effect of piperine with another main compound which should be further studied in the future.

From these results, it may be concluded that the BJK extract remedy is anti-inflammatory in its effect on pain relief, inflammation reduction, improvement in quality of daily life activity, and decreasing the modified Thai WOMAC score and the 100-meter walking times. However, there was no evidence that BJK was more efficacious than diclofenac.

According to previous clinical trial phase I study on BJK extract, the patients were assigned to take BJK extract capsule after meal three times per day [6]. The most adverse drug reactions derived from patient record form were symptoms concerning to heating and burning sensation of mouth, esophagus, and stomach. We suggested that piperine in BJK activated the transient receptor potential vanilloid type 1 (TRPV1) leading to painful and burning sensation. Although there is no current supporting evidence about activation of this receptor with piperine in BJK, several studies have demonstrated the activation of TRPV1 receptor by piperine in black pepper (*Piper nigrum* Linn.) [18–20]. It might be assumed that piperine in BJK extract can also activate the TRPV1 receptor in which omeprazole, a proton pump inhibitor, may not prevent the GI adverse effects from the BJK extract capsule intake.

This study also confirms results of previous work that NSAIDs, such as diclofenac, are well-known for drug-induced nephrotoxicity [21] because they inhibit local kidney prostaglandin that affects renal functions. And they also cause AST or ALT levels to rise because they are associated with aminotransferase elevations [22].

In studies of acute and chronic toxicity of BJK extract remedy, no liver and renal toxicity have been shown [4]. This is similar to the previous study in clinical trial phase 1 [6]. These results will be used in scientific data to support the use of the BJK extract remedy by medical doctors. The further studies of the BJK remedy extract should focus on molecular biology and the mechanism of its anti-inflammatory activity in the future. Using a larger sample size in clinical trial phase III, long term use, efficacy, and safety of dose at 100 mg/capsule/meals of the BJK remedy extract in primary osteoarthritis of knee should be also studied.

The limitation of this study was gender recruitment bias because almost outpatients who visited orthopedics department were female that relate to worldwide prevalence of the OA of knee in that time [23]. Thus 90% of female patients were recruited to both groups of this study.

In addition, this is a short-term study because of the limitation in long term use of NSAIDs and the safety of BJK extract was approved only for 1 month in clinical trial phase 1.

This is the first research report on treatment of OA patients with BJK extract and its comparison with diclofenac. This report supports using BJK remedy extract capsule to treat OA in hospitals in Thailand.

## 5. Conclusions

The BJK remedy extract showed clinical efficacy in relieving symptoms of OA knee. The anti-inflammatory effect of BJK remedy extract also showed an improvement of physical functions in daily life of OA patient. It also has no toxicity on liver and renal function and no severe adverse events of the drug. It might be an alternative choice of treatment for osteoarthritis of the knee joint. However, there was no evidence that BJK was more efficacious than diclofenac.

## Figures and Tables

**Table 1:** Medicinal plants in Benjakul remedy formulations.

Thai name	Scientific name	Voucher specimen	Part of use	Collected from
De-Plee	*Piper retrofractum *Vahl.	SKP 146160301	Fruit	Kanchanaburi
Cha-Plu	*Piper sarmentosum *Roxb.	SKP 146161901	Root	Kanchanaburi
Sa-Khan	*Piper interruptum *Opiz.	SKP 146160901	Stem	Kanchanaburi
Jayt-Moon-Plerng-Daeng	*Plumbago indica* L.	SKP 148160901	Root	Kanchanaburi
King	*Zingiber officinale* Roscoe.	SKP 206261501	Rhizome	Kanchanaburi

**Table 2:** Baseline characteristics of patients.

Data^*∗*^	BJK recipe (*n* = 42)	Diclofenac (*n* = 42)	*P*value^*∗∗*^
Female, number (%)	38 (90.5)	39 (92.9)	0.693^c^
Age; yrs, mean (SD)	61.67 (7.3)	63.5 (8.19)	0.282^t^
Weight; kg., mean (SD)	66.14 (9.48)	64.31 (10.03)	0.394^t^
Height; cm., mean (SD)	155.76 (7.12)	155.31 (8.17)	0.464^m^
BMI; Kg/m^2^, mean (SD)	27.23 (3.07)	26.62 (3.28)	0.277^m^
Visual analogue scale (VAS; mm., mean (SD))	31.02 (21.54)	36.05 (22.69)	0.159^m^
100-meter walking time; sec., mean (SD)	124.19 (24.31)	131.6 (33.56)	0.638^m^

WOMAC index score, mean (SD)			
Pain index	16.19 (7.16)	18.69 (7.06)	0.119^m^
Stiff index	5.4 (3.47)	7.48 (5.08)	0.056^m^
Physical function index	49.73 (19.93)	59.8 (26.19)	0.062^m^
Total score	71.49 (27.64)	85.68 (35.5)	0.051^m^

Laboratory data, mean (SD)			
*Blood pressure*			
Systolic (mm·Hg)	126.6 (12.82)	126.26 (17.24)	0.92^t^
Diastolic (mm·Hg)	81.9 (9.23)	78.31 (8.5)	0.067^t^
*Renal function tests*			
BUN (mg/dL)	13.24 (3.7)	13.3 (3.4)	0.941^t^
Creatinine (mg/dL)	0.71 (0.19)	0.71 (0.17)	0.865^m^
*Liver function tests*			
AST (U/L)	23.6 (10.68)	23.12 (8.27)	0.993^m^
ALT (U/L)	39.6 (12.2)	40.69 (15.1)	0.816^m^
ALP (U/L)	90.45 (18.19)	90.38 (22.6)	0.99^t^

^*∗*^Data represent mean (SD), ^*∗∗*^statistical analysis: ^t^independent two-sample Student's *t*-test, ^m^Mann-Withney  *U* test. ^c^Chi-square test.

**Table 3:** The radiographic grading at entry into the study.

Kellgren and Lawrence X-ray grade	BJK recipe (*n* = 42)	Diclofenac (*n* = 42)	*P* value^*∗*^
Grade 1	2	1	0.137^c^
Grade 2	26	18	
Grade 3	14	23	

^*∗*^Statistical analysis: ^c^chi-square test.

**Table 4:** The efficacy outcome of Benjakul recipe and diclofenac.

Data^*∗*^	Follow-up	Treatments		
		BJK recipe (*n* = 42)	Diclofenac (*n* = 42)	*P* value^*∗∗*^
Visual analogue scale (VAS) (mm.)	Day 0	31.02 (21.54)	36.05 (22.69)	0.1^m^
	Day 14	30.3 (27.22)	22.31 (20.35)^†††^	0.26^m^
	Day 28	20.97 (23.01)	19.03 (18.29)^†††^	0.99^m^

100-meter walking time (second)	Day 0	124.19 (24.31)	131.6 (33.56)	0.64^m^
	Day 14	118.35 (24.55)	120 (20.45)^††^	0.52^m^
	Day 28	119.05 (23.07)	125 (24.44)	0.24^m^

WOMAC index score				

Pain index	Day 0	16.19 (7.16)	18.69 (7.06)	0.119^m^
	Day 14	14.65 (9.37)	13.54 (7.75)^†††^	0.937^m^
	Day 28	10.38 (9.26)^†††^	10.89 (6.75)^†††^	0.383^m^

Stiff index	Day 0	5.4 (3.47)	7.48 (5.08)	0.056^m^
	Day 14	4.93 (4.15)	5.3 (3.89)^††^	0.541^m^
	Day 28	3.79 (3.84)^†††^	4.34 (3.54)^†††^	0.333^m^

Physical function index	Day 0	49.73 (19.93)	59.8 (26.19)	0.062^m^
	Day 14	45.92 (26.73)	42.79 (22.88)^†††^	0.919^m^
	Day 28	31.84 (25.09)^†††^	34.63 (22.59)^†††^	0.43^m^

Total score	Day 0	71.49 (27.64)	85.68 (35.5)	0.051^m^
	Day 14	65.79 (39.2)	60.87 (30.75)^†††^	0.927^m^
	Day 28	46.32 (37.26)^†††^	49.87 (30.09)^†††^	0.363^m^

^*∗*^Data represent mean (SD), ^*∗∗*^statistic analysis: ^m^Mann-Withney  *U* test. For all within group statistical analysis: Repeated Measure ANOVA. ^†^Significant difference from day 0 within group (*P* value < 0.05), ^††^significant difference from day 0 within group (*P* value **≤** 0.01), and ^†††^significant difference from day 0 within group (*P* value **≤** 0.001).

**Table 5:** Overall assessment of treatment evaluated at day 28.

Global assessment (point)	BJK (*n* = 39)	Diclofenac (*n* = 38)	*P*value^*∗*^
	Number (%)	Number (%)	
0: none	2 (5.1)	1 (2.6)	0.403^c^
1: mildly better	8 (20.5)	6 (15.8)	
2: moderately better	19 (48.7)	13 (34.2)	
3: much better	9 (23.1)	16 (42.1)	
4: excellent	1 (2.6)	2 (5.3)	

^*∗*^Statistical analysis: ^c^chi-square test.

**Table 6:** Adverse events of Benjakul recipe and diclofenac.

Adverse events	BJK (*n* = 42)	Diclofenac (*n* = 42)	*P*value^*∗*^
	Number (%)	Number (%)	
Stomach pain	4 (9.52)	3 (7.14)	0.693
Flatulence	1 (2.38)	2 (4.76)	0.557
Heartburn	2 (4.76)	4 (9.52)	0.397
Dry lips and throat	3 (7.14)	1 (2.38)	0.306
Palpitation	1 (2.38)	2 (4.76)	0.557

^*∗*^Statistical analysis: chi-square test.

**Table 7:** Blood pressure, renal functions, and liver functions in safety issue.

Data^*∗*^	Follow-up	Treatments		
		BJK recipe (*n* = 42)	Diclofenac (*n* = 42)	*P*value^*∗∗*^
Blood pressure				

Systolic blood pressure (normal ≤ 140 mm·Hg)	Day 0	126.6 (12.82)	126.26 (17.24)	0.92^t^
	Day 14	126.25 (15.24)	128.05 (15.28)	0.601^t^
	Day 28	122.64 (12.41)	125.22 (14.59)	0.411^t^

Diastolic blood pressure (normal ≤ 90 mm·Hg)	Day 0	81.90 (9.23)	78.31 (8.5)	0.067^t^
	Day 14	79.8 (9.07)	81.95 (12.43)	0.382^t^
	Day 28	80.26 (8.43)	80.61 (10.97)	0.875^t^

Renal functions				

Blood urea nitrogen; BUN (mg/dL) (ref.range = 7.0–18.0)	Day 0	13.24 (3.7)	13.3 (3.4)	0.941^t^
	Day 14	13.05 (3.98)	14.84 (3.67)^††^	0.041^t^
	Day 28	12.9 (3.29)	15.38 (3.82)^†^	0.003^t^

Creatinine (mg/dL) (ref.range = 0.7–1.3)	Day 0	0.71 (0.19)	0.7 (0.17)	0.865^m^
	Day 14	0.73 (0.18)	0.73 (0.17)	0.821^m^
	Day 28	0.69 (0.17)	0.73 (0.17)^†^	0.424^m^

Liver functions				

AST (U/L) (ref.range = 15–37)	Day 0	23.6 (10.68)	23.12 (8.27)	0.993^m^
	Day 14	25.28 (11.34)	28.1 (10.67)^†^	0.157^m^
	Day 28	25.03 (11.8)	26.6 (8.49)^†^	0.096^m^

ALT (U/L) (ref.range = 30–65)	Day 0	39.6 (12.2)	40.69 (15.1)	0.816^m^
	Day 14	39.65 (14.36)	45.77 (15.28)^††^	0.016^m^
	Day 28	40.33 (14.6)	44.92 (14.53)^†^	0.107^m^

ALP (U/L) (ref.range = 50–136)	Day 0	90.45 (18.19)	90.38 (22.6)	0.987^t^
	Day 14	90.95 (18.84)	97.38 (24.52)	0.194^t^
	Day 28	91.49 (19.51)	100.82 (30.53)^†^	0.113^t^

^*∗*^Data represent mean (SD), ^*∗∗*^statistic analysis: ^t^independent two-sample Student's *t*-test, ^m^Mann-Withney  *U* test. For all within group statistical analysis: Repeated Measure ANOVA. ^†^Significant difference from day 0 within group (*P* value < 0.05), ^††^significant difference from day 0 within group (*P* value ≤ 0.01), and ^†††^significant difference from day 0 within group (*P* value ≤ 0.001).
